# Remote ischemic preconditioning delays the onset of acute mountain sickness in normobaric hypoxia

**DOI:** 10.14814/phy2.12325

**Published:** 2015-03-05

**Authors:** Marc M Berger, Hannah Köhne, Lorenz Hotz, Moritz Hammer, Kai Schommer, Peter Bärtsch, Heimo Mairbäurl

**Affiliations:** 1 Department of Anesthesiology, University of HeidelbergHeidelberg, Germany; 2 Department of Anesthesiology, Perioperative and General Critical Care Medicine, Salzburg General Hospital, Paracelsus Medical UniversitySalzburg, Austria; 3 Department of Internal Medicine VII, Division of Sports Medicine, University of HeidelbergHeidelberg, Germany

**Keywords:** AMS, high altitude, oxidative stress, prevention, reactive oxygen species

## Abstract

Acute mountain sickness (AMS) is a neurological disorder occurring when ascending too fast, too high. Remote ischemic preconditioning (RIPC) is a noninvasive intervention protecting remote organs from subsequent hypoxic damage. We hypothesized that RIPC protects against AMS and that this effect is related to reduced oxidative stress. Fourteen subjects were exposed to 18 hours of normoxia (21% oxygen) and 18 h of normobaric hypoxia (12% oxygen, equivalent to 4500 m) on different days in a blinded, randomized order. RIPC consisted of four cycles of lower limb ischemia (5 min) and 5 min of reperfusion, and was performed immediately before the study room was entered. A control group was exposed to hypoxia (12% oxygen, *n* = 14) without RIPC. AMS was evaluated by the Lake Louise score (LLS) and the AMS-C score of the Environmental Symptom Questionnaire. Plasma concentrations of ascorbate radicals, oxidized sulfhydryl (SH) groups, and electron paramagnetic resonance (EPR) signal intensity were measured as biomarkers of oxidative stress. RIPC reduced AMS scores (LLS: 1.9 ± 0.4 vs. 3.2 ± 0.5; AMS-C score: 0.4 ± 0.1 vs. 0.8 ± 0.2), ascorbate radicals (27 ± 7 vs. 65 ± 18 nmol/L), oxidized SH groups (3.9 ± 1.4 vs. 14.3 ± 4.6 *μ*mol/L), and EPR signal intensity (0.6 ± 0.2 vs. 1.5 ± 0.4 × 10^6^) after 5 h in hypoxia (all *P* < 0.05). After 18 hours in hypoxia there was no difference in AMS and oxidative stress between RIPC and control. AMS and plasma markers of oxidative stress did not correlate. This study demonstrates that RIPC transiently reduces symptoms of AMS and that this effect is not associated with reduced plasma levels of reactive oxygen species.

## Introduction

Acute mountain sickness (AMS) is a syndrome of nonspecific neurologic symptoms typically experienced by nonacclimatized mountaineers within 5–12 h after arrival to altitudes >2500 m (Bartsch and Swenson 2013). The cardinal symptom is headache that is usually accompanied by anorexia, nausea, dizziness, malaise, sleep disturbance, or a combination of these symptoms (Singh et al. 1969; Bartsch and Swenson 2013). Cerebral edema due to increased capillary permeability and increased intravascular pressure have been suggested as underlying mechanisms (Willmann et al. 2014) and increased reactive oxygen species (ROS) might contribute to a capillary leak (Bailey et al. 2006), although studies using antioxidants for prevention of AMS yield controversial results (Bailey and Davies 2001; Bailey et al. 2004; Baillie et al. 2009).

Remote ischemic preconditioning (RIPC) is a noninvasive intervention capable of protecting an organ remote from the ischemic site from the damage induced by subsequent hypoxia or ischemia. Protective effects of RIPC have been found for the heart, kidney, liver, stomach, lung, and the skeletal muscle (Kanoria et al. 2007). In addition, emerging evidence suggests that the brain can be protected by RIPC (Perez-Pinzon et al. 1997; Vlasov et al. 2004; Dave et al. 2006; Jensen et al. 2011; Malhotra et al. 2011; Koch and Gonzalez 2013).

RIPC is typically induced by inflation and deflation of a standard blood pressure cuff on a limb, with several consecutive episodes of ischemia and reperfusion. The protective effects of RIPC are largely attributed to effects on vasoactive and inflammatory pathways, and several humoral mediators – including free radicals – have been implicated in mediating the beneficial effects of RIPC (Gho et al. 1996; Birnbaum et al. 1997; Kharbanda et al. 2002; Auchampach et al. 2004; Ali et al. 2007; Kanoria et al. 2007; Botker et al. 2010). Rendering a limb transiently ischemic to protect the brain might be of great clinical relevance, because this noninvasive intervention can easily been applied to humans. However, only few studies investigated the neuroprotective effect of RIPC in humans yet (Koch and Gonzalez 2013).

On the basis of the above studies, we hypothesized that RIPC would decrease the severity of AMS compared to a control group not undergoing RIPC. Postulating that a hypoxia-induced free radical damage of the blood–brain barrier is involved in the pathophysiology of AMS, we also hypothesized that RIPC would reduce oxidative stress. Therefore, we also measured plasma concentrations of ROS and antioxidants.

## Materials and Methods

### Study cohort

Fourteen (nine male, five female) healthy, nonsmoking lowlanders (age: 24 ± 1 years; body weight: 72 ± 3 kg; height: 177 ± 2 cm; body mass index: 23.3 ± 2.3; highest altitude ever reached before: 3248 ± 196 m) participated in the study. None of the participants was exposed to altitudes >2000 m within 30 days before the study and during the study period. No participant took regular medications, and none had a history of chronic headache. All subjects were encouraged to follow a low nitrate/nitrite diet on the day prior to the study days. During the study days, the participants received standardized food and beverages. The study was conducted in accordance with the Declaration of Helsinki and its current amendments and was approved by the Ethics Committee of the Medical Faculty of the University of Heidelberg (protocol number S-336/2011). Prior to the study all participants provided written informed consent.

### Study protocol

**Figure 1:**
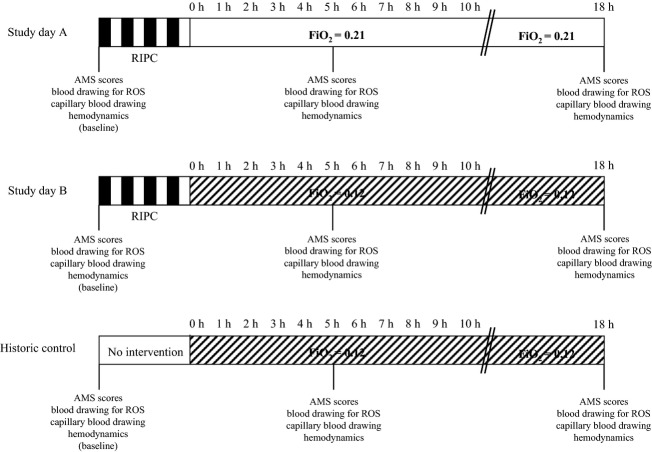
Study protocol. RIPC was performed by four 5-min cycles of lower limb ischemia interspaced with 5 min of reperfusion. Due to the nature of the intervention blinding subjects with respect to RIPC was not possible. Therefore, the data obtained in hypoxia (study day B) were compared with control data obtained in a previous study from 14 subjects that were exposed to hypoxia (FiO_2_ = 0.12) without RIPC (historic control).(Schommer et al. 2012) AMS scores were assessed with the Lake Louise score and the AMS-C score of the Environmental Symptom Questionnaire. Venous blood samples were drawn from a catheter inserted into a cubital vein for the analyses of reactive oxygen species (ROS), and capillary blood samples were taken from the hyperemic ear lobe for blood gas measurements.

Each participant was exposed to 18 h of normoxia (21% oxygen) and 18 h of normobaric hypoxia (12% oxygen, rest N_2_, equivalent to an altitude of 4500 m) in randomized order. The exposure to normoxia was performed to blind the subjects with respect to the ambient oxygen concentration, and thus to avoid a bias on the measured AMS scores. To avoid acclimatization effects the two study days were at least 4 weeks apart. RIPC was performed on both study days following an identical protocol. Due to the nature of the intervention blinding of the subjects to RIPC was not possible. Therefore, the data obtained in hypoxia were compared with data from 14 healthy, nonsmoking subjects (10 men, four women; age: 29 ± 2 year; body weight: 70 ± 3 kg; height: 176 ± 2 cm; body mass index 22.4 ± 2.0; highest altitude ever reached before: 4098 ± 213 m) that were exposed to an identical protocol of normobaric hypoxia (12% oxygen, 18 h) without RIPC in a previous study (Schommer et al. 2012). Also in this study population no participant took regular medications, and none had a history of chronic headache. Figure1 summarizes the study design.

RIPC was performed in normoxia in supine position 45 min before the hypoxia room was entered. The RIPC protocol consisted of four 5-min cycles of lower limb ischemia interrupted by 5 min of reperfusion. Ischemia was induced by automatically inflating a blood pressure cuff to 200 mmHg on both thighs which in all cases was at least 45 mmHg above the measured systolic arterial blood pressure.

Before the intervention (baseline) and at 5 and 18 h capillary blood samples were taken from the ear lobe treated with vasodilator cream (Finalgon) for the measurement of blood gases and pH (Siemens Rapidpoint 400/405; Bayer Diagnostics, Sudbury, UK). At these time points heart rate, blood pressure, and severity of AMS were assessed, and blood samples for the measurement of markers of oxidative stress were collected from a cubital vein. In this study and in the control study, the protocol for blood processing, AMS assessment, and hemodynamic measurements were exactly the same.

### Assessment of acute mountain sickness (AMS)

The severity of AMS was evaluated by clinical examination and was quantified using the Lake Louise scoring protocol and the AMS-C score of the Environmental Symptom Questionnaire as described previously (Berger et al. 2009; Schommer et al. 2012). Subjects were classified as AMS positive with an AMS-C score ≥0.70 in combination with a Lake Louise score ≥5 when headache was present. If only one of both scores was positive the subject was classified as non-AMS.

### Metabolic analyses

For L-ascorbate measurements, 900 *μ*L of 5% metaphosphoric acid was added to 100 *μ*L K^+^-EDTA-plasma and assayed by fluorimetry based on the condensation of dehydroascorbic acid with 1,2-phenylenediamine as described previously (Bailey et al. 2010). For the measurement of the ascorbate free radical, 20 *μ*L of plasma was injected into a high-sensitivity multiple-bore sample cell (AquaX; Bruker Instruments Inc, Billerica, MA) housed within a TM_110_ cavity of an EPR spectrometer (nOxyscan, Noxygen Science Transfer & Diagnostics GmbH, Germany). Quantification of the radical concentration was achieved by double integration of the spectra (Microcal Origin software) using a reference solution of stable free radical 3-carboxyl-2,2,5,5-tetramethyl-3-pyrroline-1-oxyl as a calibration standard. Oxidized sulfhydryl (SH) groups were analyzed by spectrophotometry as described previously (Goraca et al. 2013).

### Statistics

Normal distribution of the data was tested using the Kolmogorov–Smirnov test. Data obtained periodically throughout the experiment, such as parameters of oxidative stress, were analyzed using a two-factor (factor A: intervention × factor B: time) repeated measures ANOVA with post hoc Holm–Sidak test. The relationship between pairs of variables was expressed with the Pearson correlation coefficient. Differences in the incidence of AMS were analyzed with the Fisher and Yates Test. Plasma markers of ROS in normoxia were analyzed with a one-way repeated measure ANOVA and a Holm–Sidak post hoc test. Data are expressed as mean values ± SE. A *P* value of ≤0.05 was considered significant. Statistics were performed using the SigmaStat software package (SPSS, Chicago, IL).

## Results

### Effect of RIPC on AMS

**Figure 2:**
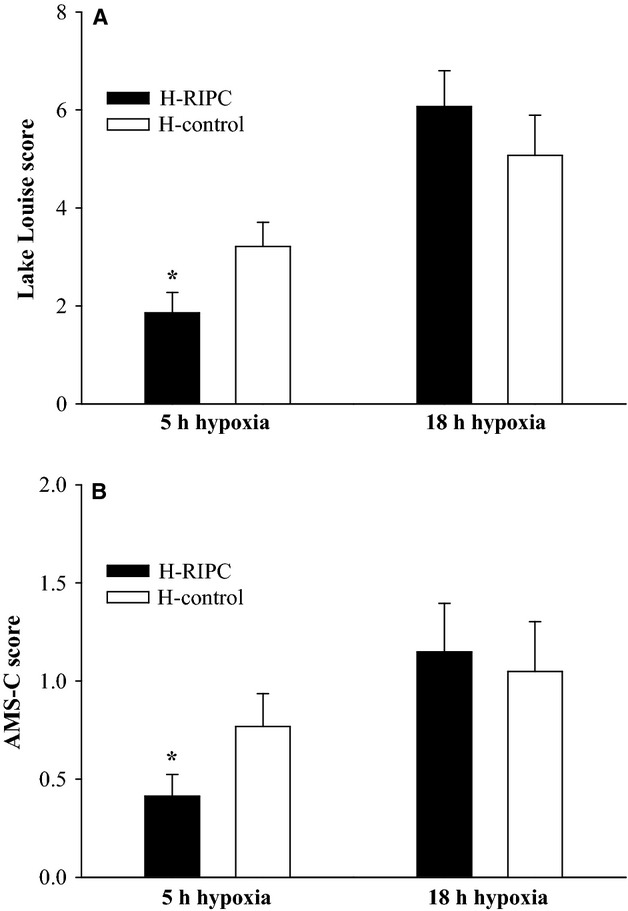
(A) Severity of AMS as evaluated by the Lake Louise score, and by the AMS-C score of the Environmental Symptom Questionnaire (B). A Lake Louise score of ≥5 points and an AMS-C score of ≥0.7 points represents AMS. H-RIPC: group exposed to hypoxia undergoing RIPC. H-Control: group exposed to hypoxia without RIPC. *P < 0.05 vs. H-Control.

As published previously (Schommer et al. 2012), exposure to hypoxia without RIPC caused an increase in the Lake Louise score and the AMS-C score to 3.2 ± 0.5 and 0.8 ± 0.2 points after 5 h, and to 5.1 ± 0.8 and 1.1 ± 0.3 points after 18 h, respectively (Fig.2, all *P* < 0.01).

RIPC significantly reduced AMS severity at 5 h of hypoxia as evaluated by the Lake Louise score and the AMS-C score, respectively (Fig.2; *P* < 0.05 vs. nonpreconditioned control). However, after 18 h in hypoxia AMS severity was not different between the RIPC and the nonpreconditioned control group (Fig.2). In line with these findings the incidence of AMS tended to be lower in the RIPC group at 5 h (RIPC: 7%, control: 21%; *P* = 0.29), but not at 18 h (43% in both groups). Both AMS scores, that is, the Lake Louise score and the AMS-C score, showed a positive correlation (*R* = 0.86, *P* < 0.001; not shown).

When RIPC was performed preceding normoxia both the Lake Louise score and the AMS-C score remained low (≤0.3 and ≤0.05, respectively; not significant vs. baseline at all time points; not shown), indicating that exposure to the experimental setting (RIPC, laboratory) itself did not induce AMS-like symptoms.

### Effect of RIPC on oxidative stress

Table1 compares the plasma levels of markers of oxidative stress in hypoxia. After 5 h of hypoxia the EPR signal intensity, and the plasma concentrations of oxidized SH groups and of ascorbate free radicals were significantly lower in the RIPC group than in the nonpreconditioned hypoxic control group (all *P* < 0.05; Table1). After 18 h in hypoxia, there was no statistical significant difference in ROS between both study groups. RIPC was also associated with a significant decrease in the plasma concentration of the antioxidant L-ascorbate after 5 h in hypoxia (*P* < 0.05; Table1).

**Table 1:** Plasma concentrations of the ascorbate free radical, of oxidized SH groups, and EPR signal intensity during hypoxia (12% oxygen). The baseline values represent the concentration before any intervention. Values are means ± SE

	Baseline	Normobaric hypoxia		*P* value		
		5 h	18 h	Time	Intervention	Time × Intervention
Ascorbate free radical (nmol/L)						
H-RIPC	47.5 ± 18.4	27.0 ± 7.4*	24.2 ± 6.8	0.220	0.03	0.316
H-Control	55.8 ± 14.6	65.0 ± 18.2	38.0 ± 10.5			
EPR signal intensity						
H-RIPC	1.09 ± 0.42	0.62 ± 0.17*	0.55 ± 0.16	0.221	0.03	0.317
H-Control	1.28 ± 0.33	1.49 ± 0.42	0.87 ± 0.24			
Oxidized SH groups (*μ*mol/L)						
H-RIPC	7.4 ± 3.0	3.9 ± 1.4*	4.3 ± 1.3	0.366	0.02	0.319
H-Control	10.9 ± 4.0	14.3 ± 4.6	6.6 ± 2.2			
L-ascorbate (*μ*mol/L)						
H-RIPC	58.6 ± 22.7	33.3 ± 9.1*	29.9 ± 8.4	0.220	0.04	0.317
H-Control	68.9 ± 18.0	80.2 ± 22.5	46.9 ± 12.9			

* *P* < 0.05 vs. H-Control at the same time point.

In normoxia RIPC decreased plasma levels of ascorbate free radicals, of EPR signal intensity, and of L-ascorbate at 5 and 18 h, respectively (*P* < 0.05; Table2).

**Table 2:** Plasma concentrations of the ascorbate free radical, of oxidized SH groups, and EPR signal intensity during normoxia. The baseline values represent the concentration before any intervention. Values are means ± SE

	Baseline	5 h	18 h
Ascorbate free radical (nmol/L)	51.3 ± 13.0	33.1 ± 12.1*	34.6 ± 9.3*
EPR signal intensity	1.17 ± 0.30	0.76 ± 0.28*	0.79 ± 0.21*
Oxidized SH groups (*μ*mol/L)	8.5 ± 2.3	5.2 ± 2.0*	5.5 ± 1.5*
L-ascorbate (*μ*mol/L)	63.1 ± 15.9	40.9 ± 14.9*	42.6 ± 11.5*

* *P* < 0.05 vs. baseline.

### Effect of RIPC on oxygenation

As anticipated, hypoxic exposure was associated with marked hypoxemia and respiratory alkalosis (Table3). Upon hypoxia, heart rate significantly increased (*P* < 0.001), whereas blood pressure remained unchanged (Table3). There was no significant difference in these values between the RIPC and the nonpreconditioned hypoxic control group.

**Table 3:** Blood gas analysis and hemodynamics. The baseline values represent the concentration before any intervention. Values are means ± SE

	Baseline	Normobaric hypoxia		*P* value		
		5 h	18 h	Time	Intervention	Time × Intervention
pO_2_ (mmHg)						
H-RIPC	86 ± 2.4	38 ± 1.3*	38 ± 1.2*	<0.001	0.133	0.191
H-Control	80 ± 2.2	36 ± 1.2*	37 ± 1.1*			
pCO_2_ (mmHg)						
H-RIPC	38 ± 1.0	31 ± 0.7*	30 ± 0.8*	<0.001	0.171	0.448
H-Control	39 ± 0.9	34 ± 0.8*	31 ± 0.8*			
ph						
H-RIPC	7.42 ± 0.01	7.47 ± 0.01*	7.48 ± 0.01*	<0.001	0.382	0.879
H-Control	7.43 ± 0.00	7.48 ± 0.01*	7.47 ± 0.01*			
Systolic blood pressure (mmHg)						
H-RIPC	117 ± 3.2	117 ± 3.7	119 ± 3.3	0.213	0.193	0.866
H-Control	121 ± 2.9	120 ± 3.6	125 ± 3.6			
Diastolic blood pressure (mmHg)						
H-RIPC	76 ± 2.0	75 ± 3.0	76 ± 2.9	0.815	0.568	0.342
H-Control	72 ± 1.9	76 ± 3.3	71 ± 3.3			
Heart rate (beats/min)						
H-RIPC	65 ± 1.8	83 ± 3.1*	84 ± 4.1*	<0.001	0.453	0.06
H-Control	68 ± 3.2	76 ± 3.6*	81 ± 4.0*			

H-RIPC, group exposed to hypoxia after RIPC; H-Control, group exposed to hypoxia without RIPC; pO_2_, capillary oxygen tension; pCO_2_, capillary carbon dioxide tension.

* *P *<* *0.05 vs. baseline in the respective condition.

## Discussion

Several studies have shown that RIPC can protect the brain from a hypoxic or ischemic damage (Perez-Pinzon et al. 1997; Vlasov et al. 2004; Dave et al. 2006; Jensen et al. 2011; Malhotra et al. 2011; Koch and Gonzalez 2013). This protection is associated with alterations of vasoactive and inflammatory pathways (Gho et al. 1996; Birnbaum et al. 1997; Kharbanda et al. 2002; Auchampach et al. 2004; Ali et al. 2007; Kanoria et al. 2007; Botker et al. 2010), both of which play a major role in the pathophysiology of AMS (Roach and Hackett 2001; Basnyat and Murdoch 2003; Ainslie and Subudhi 2014). The present prospective, randomized, and controlled study shows for the first time that RIPC, induced by transient lower limb ischemia, reduced symptoms of AMS after 5 h but not after 18 h of exposure to normobaric hypoxia at an FiO_2_ corresponding to an altitude of 4500 m.

### Time course of RIPC-induced AMS reduction

The lack of RIPC-induced protection against AMS after 18 h may be explained by the typical biphasic protection induced by a preconditioning stimulus: An early protective phase develops within a few minutes after the preconditioning stimulus and lasts for several hours. A second late phase becomes apparent after 12–72 h and elicits a maximum of protection several days after the preconditioning stimulus (Bolli 2000; Kanoria et al. 2007; Narayanan et al. 2013). Both phases are separated by a window where no protection occurs (Bolli 2000; Loukogeorgakis et al. 2005). The mechanisms for these two phases are completely different: While the early phase is assumed to be caused by rapid posttranslational modification of preexisting proteins (e.g., protein kinase C and mitogen-activated protein kinases), the late phase is most likely caused by the synthesis of new protective proteins, such as for example Src protein tyrosine kinases, and Janus-activated kinases (Bolli 2000, 2007), which explains the different time courses of these phenomena. Thus, it is conceivable that the reduced AMS scores at 5 h were due to a protective effect of the early phase, and that the lack of protection after 18 h was due to the intermittent nonprotection phase. Another possibility is that RIPC induced a delayed onset of AMS rather than causing a biphasic protection, suggesting that RIPC has no clinically relevant effect on AMS, which is usually most prominent after the first night at a higher altitude (Bartsch and Swenson 2013). One could speculate that individuals might have been protected again upon longer exposure, but observation periods longer than 18 h have not been investigated yet, and need to be addressed in future studies. Since subjects cannot be blinded to the application of RIPC, we cannot exclude that a placebo effect prevented perception of milder symptoms of AMS in the early hours and accounts for the delayed onset of AMS after RIPC. However, to avoid bias on the measured AMS scores we exposed the RIPC group twice in randomized, blinded order to normoxia and hypoxia, respectively, and compared the results obtained in hypoxia with the results from subjects that were exposed to an identical protocol of normobaric hypoxia without RIPC in a previous study.

Although the RIPC protocol applied in this study (four 5-min cycles of lower limb ischemia interspaced with 5 min of reperfusion) has been demonstrated to confer protection in different species, including humans, pigs, rats, and mice (Kharbanda et al. 2002; Li et al. 2004; Kristiansen et al. 2005; Cheung et al. 2006), it is possible that other RIPC protocols than the one we applied are more effective against AMS. The efficacy of a RIPC stimulus is protocol specific (depending on the organ targeted at and on species) (Kanoria et al. 2007) and it is conceivable that increasing the number and/or duration of RIPC applications prior to the hypoxic exposure, or in repeating the treatment for many days prior to the exposure, could improve outcome.

### Role of oxidative stress in RIPC and AMS

The mechanisms by which RIPC exerts its protective effects have not been fully elucidated, but evidence suggests that ROS are involved in this process by triggering vasoactive and inflammatory pathways (Gho et al. 1996; Birnbaum et al. 1997; Kharbanda et al. 2002; Kanoria et al. 2007; Botker et al. 2010). In this study, RIPC was associated with decreased ROS levels at all time points in normoxia. Moreover, after 5 h in hypoxia ROS plasma levels were significantly lower in the RIPC group compared to nonpreconditioned hypoxic controls. Our findings that the decrease in ROS was paralleled by a decrease in L-ascorbate, indicate that an increased antioxidant plasma capacity was not responsible for the RIPC-induced reduction in ROS. This is in line with a previous observation in pigs, showing that late preconditioning protected against myocardial stunning without being related to antioxidant defenses (Tang et al. 1997). A recent study by Rassaf et al. (2014) in mice found that circulating nitrite derived from shear stress-dependent stimulation of endothelial nitric oxide synthase contributes to reduced ROS formation upon RIPC. In this study, nitric oxide and its metabolites, for example, nitrite/nitrate, have not been measured, and the pathway(s) underlying the RIPC-induced reduction in ROS remain(s) speculative.

The lower ROS levels in the RIPC group after 5 h in hypoxia were accompanied by decreased AMS scores. While this observation supports the concept that ROS may play a role in the pathophysiology of AMS, our findings after 18 h speak against this hypothesis because ROS plasma levels remained decreased in both groups, although AMS scores increased. The dissociation between ROS and the severity of AMS might suggest that an increased oxidative stress is not involved in the pathophysiology of AMS. This interpretation is supported by results from a double-blind, randomized placebo controlled trial, showing that oral antioxidant supplementation did not prevent AMS (Baillie et al. 2009). However, it remains unclear how well systemic plasma levels of ROS reflect ROS metabolism of the brain. It is conceivable that local transitory ROS generation initiates a local, cerebral inflammatory/vasoactive cascade that is not reflected in the systemic circulation, and that the markers measured in this case reflect phenomena occurring in other tissues. In this context, one study by Bailey and Davies has to be mentioned, showing a moderately beneficial effect of the antioxidant vitamin C on AMS (Bailey and Davies 2001). The different outcome of both interventional studies on the effect of antioxidants on AMS might be explained in part by different study settings. The negative study involved rapid passive ascent from sea level to 5200 m in 5 days including a staging of 4 days at 3800 m and carries a high risk for AMS, whereas the positive study involved a slow ascent from 1400 m to 5180 m in 10 days resulting in an average ascent rate of 378 m/day, which reduces the risk for AMS considerably. The dissociation between ROS plasma levels and AMS after 18 h further indicates that the effect of RIPC on AMS after 5 h was not due to an effect on ROS. This is in line with a clinical study showing that RIPC decreased ROS and attenuated intestinal and pulmonary injury in patients undergoing abdominal aortic aneurysm repair, but that also did not establish a causal relationship between protection and ROS (Li et al. 2013). However, it is likely that multiple pathways for signal transduction exist in the circulation and contribute to RIPC-induced protection.

### Limitations

Due to the nature of the procedure, it was not possible to blind subjects to the intervention of RIPC. Therefore, we performed RIPC on one study day where subjects were exposed to normoxia and on one study day with exposure to hypoxia, and blinded the subjects with respect to the ambient oxygen concentration. The results obtained in hypoxia were then compared with the findings obtained in hypoxia during an earlier study (Schommer et al. 2012) where subjects were exposed to the same hypoxia protocol without RIPC (see Fig.1). This design was chosen to obtain the most objective data regarding AMS scores. The disadvantage of this design was that data were obtained from two distinct study populations, whose anthropometric data, however, were similar. Thus, innate differences between the two study groups cannot be excluded but are unlikely to significantly affect the results of the present study. As subjects could not be blinded to the application of RIPC, we cannot exclude that a placebo effect prevented perception of mild symptoms of AMS in the early hours and accounts for the delayed onset of AMS after RIPC.

The baseline values of ROS were slightly (*P*-values between 0.5 and 0.7) higher in the control group not undergoing RIPC, what might have affected the difference after 5 h. However, the data show that in the first 5 h plasma levels of ROS were contrarily regulated in the RIPC group (values decreased) compared to the control group (values increased). This observation is in line with several other studies, for example, (Li et al. 2013). Whether the decrease in ROS is the mechanism underlying RIPC-induced protection remains, however, unclear from these data.

## Conclusion

This study demonstrates that RIPC causes a significant reduction of AMS symptoms 5 h after exposure. This effect is not associated with a reduction of ROS plasma levels. RIPC did, however, not reduce the incidence of AMS after 18 h despite a decrease of plasma levels of ROS. Before recommendations for mountaineering can be deduced from our results, studies lasting longer than 18 h are necessary for testing whether RIPC merely delays the onset of AMS or whether a biphasic pattern with a delayed second protective phase after 24 h as suggested by Bolli (Bolli 2000) accounts for the observed results.
